# *In vitro* culture of isolated primary hepatocytes and stem cell-derived hepatocyte-like cells for liver regeneration

**DOI:** 10.1007/s13238-015-0180-2

**Published:** 2015-06-19

**Authors:** Chenxia Hu, Lanjuan Li

**Affiliations:** Collaborative Innovation Center for Diagnosis and Treatment of Infectious Diseases, State Key Laboratory for Diagnosis and Treatment of Infectious Diseases, School of Medicine, First Affiliated Hospital, Zhejiang University, Hangzhou, 310006 China

**Keywords:** liver regeneration, primary hepatocyte, stem cell, hepatocyte-like cell, in vitro culture

## Abstract

Various liver diseases result in terminal hepatic failure, and liver transplantation, cell transplantation and artificial liver support systems are emerging as effective therapies for severe hepatic disease. However, all of these treatments are limited by organ or cell resources, so developing a sufficient number of functional hepatocytes for liver regeneration is a priority. Liver regeneration is a complex process regulated by growth factors (GFs), cytokines, transcription factors (TFs), hormones, oxidative stress products, metabolic networks, and microRNA. It is well-known that the function of isolated primary hepatocytes is hard to maintain; when cultured *in vitro*, these cells readily undergo dedifferentiation, causing them to lose hepatocyte function. For this reason, most studies focus on inducing stem cells, such as embryonic stem cells (ESCs), induced pluripotent stem cells (iPSCs), hepatic progenitor cells (HPCs), and mesenchymal stem cells (MSCs), to differentiate into hepatocyte-like cells (HLCs) *in vitro*. In this review, we mainly focus on the nature of the liver regeneration process and discuss how to maintain and enhance *in vitro* hepatic function of isolated primary hepatocytes or stem cell-derived HLCs for liver regeneration. In this way, hepatocytes or HLCs may be applied for clinical use for the treatment of terminal liver diseases and may prolong the survival time of patients in the near future.

## INTRODUCTION

Viral hepatitis, fatty liver disease, drug-induced liver injury, liver cirrhosis, hepatic carcinoma, and other liver diseases can cause acute or chronic liver failure. Approximately 10% of patients with liver disease succumb to their condition while waiting for liver sources each year (Kim et al., 2006).

Liver transplantation was once the only therapeutic option for patients with end stage liver diseases, and its clinical use was limited due to limited donor availability, surgical injuries, a high incidence of surgical complications and the high cost of the treatment (Duan et al., 2013). Later, cell transplantation and artificial liver support emerged as effective methods for compensation of lost liver function and increased the survival rate of patients; however, these two methods are also limited by the availability of effective cell sources and equipment. The inability of hepatocytes to proliferate *in vitro* and the severely inadequate supply of hepatocytes due to donor shortage are still the main problems for primary human hepatocyte-based treatments. Stem cells have been proposed as an ideal cell source because they have potent self-renewal, low immunogenicity, and the capacity to differentiate into various cell types. Furthermore, they can generate unlimited hepatocytes with incomplete function (Sancho-Bru et al., 2009) that are generally defined as hepatocyte-like cells (HLCs). HLCs can be derived from multiple stem cell types, such as embryonic stem cells (ESCs), induced pluripotent stem cells (iPSCs), hepatic progenitor cells (HPCs), and mesenchymal stem cells (MSCs). Therefore, it is crucial to develop robust methods for differentiating stem cells into mature hepatocytes *in vitro* for clinical use.

Here, we present an overview of isolated primary hepatocytes and stem cell-derived HLCs used for liver regeneration and describe how the *in vitro* environment in which they are cultured is continuously being optimized to mimic *in vivo* conditions and maintain hepatic function. The main disadvantages, histologic origin, 3D, and co-culture environment for *in vitro* culture of isolated hepatocytes or stem cell-derived hepatocytes were demonstrated in Table 1. Optimization of *in vitro* culturing of functional hepatocytes will solve the issues of limited cell numbers and limited function, and sufficient numbers of functional hepatocytes will be used to promote liver regeneration directly or indirectly.

**Table 1:** Main disadvantages, histologic origin, 3D, and co-culture environment for *in vitro* culture of isolated hepatocytes or stem cell-derived hepatocytes

Cell type	Main disadvantages	Histologic origin	3D environment	Co-culture with other cells
Isolated primary hepatocyte	Hard to maintain hepatic function	Liver	Collagen sandwich, chitosan-hyaluronic acid polyelectrolyte multilayer (Kim and Rajagopalan, 2010), matrigel layer(Sellaro et al., 2010), hydroxyethylmethacrylate and ethoxyethylmethacrylate copolymers (Marekova et al., 2013)	3T3-J2 fibroblasts (Cho et al., 2008), bone marrow mesenchymal stem cells (Marekova et al., 2013), human adipose-derived stem cells (No da et al., 2012)
ESC	Carry problems of ethics and immunorejection	Inner mass cells or primordial germ cells	3D spheroid culture system, rotating bioreactor, hollow fiber (Subramanian et al., 2014), biodegradable polymer scaffold (Wang et al., 2012), type I collagen and Swiss 3T3 cell sheets (Nagamoto et al., 2012)	STO feeder cells, MLSgt20 cells, HSC (Ishii et al., 2010),HepG2 cells (Lee et al., 2009), xeno-free extracellular matrix (Farzaneh et al., 2014)
iPSC	Create chimeras by germ line transmission and tetraploid complementation	Skin and nucleated blood cells and other terminally differentiated cells	Hollow fiber/organoid (Amimoto et al., 2011), micro-cavitary hydrogel (MCG) system (Lau et al., 2013), type I collagen and Swiss 3T3 cell sheets (Nagamoto et al., 2012), multi-component hydrogel fibers (Du et al., 2014)	Bone marrow mesenchymal stem cells (Mobarra et al., 2014), endothelial cells (Du et al,. 2014), liver non-parenchymal cell line TWNT-1 (Javed et al., 2014)
HPC	Lack of sources	Liver	Biomatrix scaffolds (Wang et al., 2011), 3D collagen gel matrix, fibroblast feeder layer culture system (Lazaro et al., 1998)	
MSC		Bone marrow, adipose, placenta, umbilical cord, amniotic membrane and other tissues	Nanofibers and alginate scaffolds (Piryaei et al., 2011), 3D matrixes of poly (ethylene glycol)-b-poly(l-alanine) thermogel (Kim et al., 2014), bioartificial liver system (Yang et al., 2013)	Hepatoma-derived C3A cells (Yang et al., 2013), cystic fibrosis airway epithelial cells (Paracchini et al., 2012)

## NATURE OF LIVER REGENERATION

The liver serves as a major storage site of glycogen and vitamin A and is one of only a few organs in adults that are capable of regeneration. Normal mature hepatocytes and cholangiocytes stay in the G_0_ phase of the cell cycle, exhibit a quiescent phenotype and show minimal turnover, but in response to partial hepatectomy (PH), they can undergo cell proliferation to compensate for the lost cells, a process called liver regeneration. However, severe damage caused by liver diseases can significantly diminish the proliferative ability of these cells and, thus, their liver regeneration ability. When that is the case, liver tissue transplantation may be required (Samuel et al., 2011).

Spontaneous liver tissue regeneration (Fujiyoshi and Ozaki, 2011) is achieved by a complex interactive network consisting of liver cells (hepatocytes, kupffer cells, sinusoidal endothelial cells, hepatic stellate cells, and stem cells) and extrahepatic organs (the thyroid gland, adrenal glands, pancreas, duodenum, and autonomic nervous system). Growth factors (GFs), transcription factors (TFs), cytokines, hormones, oxidative stress products, metabolic networks, and microRNA are essential for liver regeneration to proceed in an optimal manner to gain adequate hepatic mass (Mao et al., 2014). Mitogenic GFs override the G_1_ restriction point and promote hepatocytes to transit into S phase. The restoration of liver volume depends on hepatocyte proliferation, which includes initiation, proliferation, and termination phases.

After PH, more than 100 immediate early genes are activated by TFs that are latent in the quiescent liver. Interleukin (IL)-6 (Li et al., 2001), lipopolysaccharide (Cornell et al., 1990), C3a, and C5a (Strey et al., 2003) can initiate the cytokine cascade and trigger liver regeneration. Nuclear factor-kB (Deng et al., 2009), calcitonin gene-related peptide (Mizutani et al., 2013), caspase recruitment domain-containing protein 11, zinc finger protein 490 (Nygard et al., 2012), and heat shock protein 70 (Wolf et al., 2014) contribute to the early phase of successful liver regeneration. Pituitary hormone prolactin administration directly or indirectly increases the number of proliferating cells during the priming stage of hepatectomy, which causes an increase in the binding activity of several TFs involved in cell proliferation, liver-specific differentiation, and the maintenance of energetic metabolism (Olazabal et al., 2009). Furthermore, the process of liver regeneration involves a general reduction of the levels of many coagulation cascade proteins (Tatsumi et al., 2011). Auxiliary mitogens include norepinephrine, vascular endothelial growth factor, insulin, bile acids, serotonin, complement, leptin, estrogens, fibroblast growth factor (FGF)-1, FGF-2, and IL-4 are also indispensable for the hepatocyte cell cycle (DeAngelis et al., 2012; Michalopoulos, 2010). Vitamin D3 upregulated protein-1 regulates proliferative signaling during liver regeneration by altering the activation of genes involved in the extracellular signal-regulated kinase 1/2 and AKT signaling pathways (Kwon et al., 2011). Growth hormone signaling molecules (Zerrad-Saadi et al., 2011) and the transcription factor E2F2 (Delgado et al., 2011) are also key regulators of the cell cycle. Activation of Notch, a signaling pathway that mediates lineage segregation during liver development, is sufficient to reprogram hepatocytes into biliary epithelial cells (Yanger et al., 2013). FGF15 is an essential mediator of the liver growth promoting effects of bile acids and is necessary to maintain liver growth homeostasis (Uriarte et al., 2013). P-element-induced wimpy testis interacting RNAs exert regulatory functions on the cell genome and transcriptome (Rizzo et al., 2014). Hepatic non-parenchymal cells play a time-dependent regulatory role (Nejak-Bowen et al., 2013). Nogo-B, also known as reticulon 4B, promotes liver fibrosis and cirrhosis by facilitating the TGF-b signaling pathway in activated hepatic stellate cells (HSCs) and facilitates hepatocyte proliferation and liver regeneration (Gao et al., 2013). The termination of liver regeneration is a complex process that is affected by integrin-mediated signaling. The return of HGF and TGF-b to their baseline levels (Michalopoulos, 2010) and activation of mitogen-activated protein kinase kinase-4 (Wuestefeld et al., 2013) can cause complete termination of liver regeneration.

## *IN VITRO* CELLS WITH HEPATIC FUNCTION

**Figure 1:**
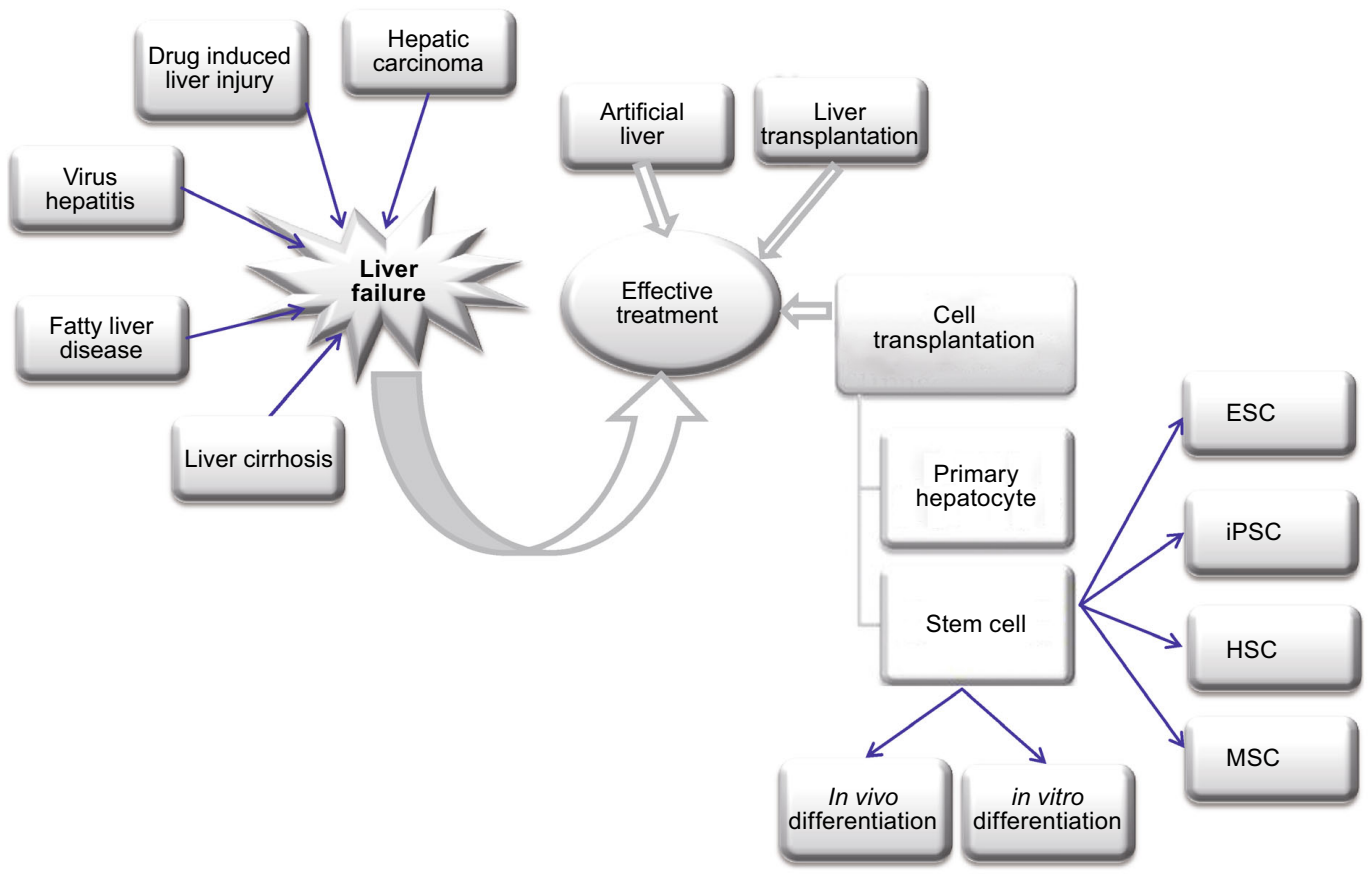
Liver regeneration is a rapid and well-coordinated process that requires contributions from multiple cell populations

Liver regeneration can proceed through two different mechanisms: replacement of lost tissue with cell types of phenotypic fidelity; and replacement of tissue by activation of transdifferentiation pathways originating from facultative stem cells. Liver regeneration is a rapid and well-coordinated process that requires contributions from multiple cell populations (Fig. 1).

### Primary hepatocytes

The liver is primarily composed of two epithelial cell lineages, namely hepatocytes and cholangiocytes, which originate from hepatoblasts during fetal development. Hepatocytes are the predominant cell type in the liver under nonpathological conditions. Isolated primary human hepatocytes are currently the gold standard for *in vitro* drug screening because they express the entire complement of hepatic drug metabolizing enzymes and transporters. In spite of their prolific growth ability *in vivo*, attempts to proliferate adult hepatocytes *in vitro* have been less successful. It has taken a long time to optimize the hepatocyte culture conditions to allow them to grow steadily *in vitro*.

Once plated in a monolayer, primary hepatocytes typically undergo progressive dedifferentiation, which is reflected at the level of the drug transporters and the dramatic loss in the phenotypic characteristics of the cells. Specifically, hepatocytes and liver sinusoidal endothelial cells dedifferentiate within 72 h when cultured as monolayers *in vitro*. Conventional approaches to counteract this dedifferentiation aim at reestablishing the natural hepatocyte microenvironment *in vitro* and include reintroduction of an extracellular matrix (ECM) backbone (Skardal et al., 2012), addition of differentiation promoting soluble compounds to the culture medium, and boosting of homotypic hepatocyte interactions or cocultivation of hepatocytes with other cell types. However, the use of this approach is limited by the availability of reproducible sources of hepatocytes.

The ability of EGF to induce DNA synthesis in primary hepatocytes was first demonstrated in 1976. Thereafter, many researchers have tried to determine the essential factors for triggering hepatic regeneration. E-cadherin is required for hepatocyte spheroid formation and may be responsible for protecting hepatocytes from a novel form of caspase-independent cell death (Luebke-Wheeler et al., 2009). Culturing of rat liver sinusoidal endothelial cells in a layered three-dimensional configuration, with the layers separated by a chitosan-hyaluronic acid polyelectrolyte multilayer, resulted in enhanced heterotypic cell-cell interactions, which led to improvements in hepatocyte function (Kim and Rajagopalan, 2010). PuraMatrix, a well-defined synthetic peptide that can self-assemble into an interweaving nanofiber scaffold to form a hydrogel, is an attractive system for generating hepatocyte spheroids that quickly restore liver function after seeding (Wang et al., 2008). More recently, hepatocytes sandwiched between matrigel layers were reported to have stable function. Despite their advantages, collagen and matrigel sandwich cultures do not provide the complex multi-cellular environment found *in vivo*, and co-cultures do not mimic the layered liver architecture (Sellaro et al., 2010). Current techniques that maintain hepatocellular function *in vitro* with different biomaterials and geometries exhibit a low cell density and functional capacity per unit volume.

### Stem cell-derived hepatocytes

Stem cells have the potential for numerous biomedical applications, including therapeutic cell replacement to repair damaged body organs, as tools for studying genetic defects and testing drugs, and as models for studying cell differentiation and early development. HLCs can be derived from ESCs (Subramanian et al., 2014), iPSCs (Amimoto et al., 2011), HPCs (Wang et al., 2011), and MSCs (Piryaei et al., 2011), which have the capacity for unlimited proliferation and multilineage differentiation. However, there are certain requirements for the generation of HLCs: (a) an unlimited source of initial cell material is needed to ensure routine large-scale generation of the required cells; (b) the generated cultures should be reproducible in terms of their hepatic-like functionality; and (c) the established system should allow for highly efficient selection of hepatocytes.

#### *Embryonic stem cell-derived hepatocytes* (*ESC-Heps*)

ESCs are derived from the inner cell mass of the fertilized egg, which is pluripotent, and can be cultured indefinitely in an undifferentiated state and have the potential to differentiate into three germ layer cell types. They have been shown to give rise to functional hepatocytes that effectively integrate into and replace injured parenchyma in many devastating liver diseases. Definitive endoderm (DE) cells are the precursors of both the liver and pancreas, and they have to be induced to undergo hepatic and pancreatic differentiation (Murry and Keller, 2008). Moreover, the expression of alpha fetal protein (AFP), albumin (ALB), and a biliary molecular marker appear sequentially, suggesting the differentiation of ESCs recapitulates the normal developmental processes of the liver. There are some issues with these the current differentiation protocols, including spontaneous differentiation, low yield, the presence of undefined and xenogenetic compounds, necessity of cell sorting for specific cell lineages, considerable enzymatic stress during repeated culture (Haque et al., 2011) and cellular heterogeneity in the culture (Nagaoka et al., 2008). Terminally specified cells of a certain lineage represent only a minor cell fraction of the differentiating ESCs in culture. Therefore, large-scale production of highly purified cell lineages of interest is the principle task of the ESC-based approach to regenerative medicine. Potential therapeutic applications of ESC-Heps are limited by their relatively low output in differentiating ESC cultures and by the danger of contamination with tumorigenic, undifferentiated ESCs. Furthermore, the use of ESCs typically causes ethical and immunorejection issues, and the elimination of these issues is critical for stem cell transplantation therapies to be effective.

Many studies have made substantial contributions to the differentiation of ESCs into hepatocytes by continuously improving inducers of differentiation and optimizing their combinations and sequences (Li et al., 2010b; Liu et al., 2010b; Wang et al., 2012; Zhang et al., 2013b). Most induction methods try to imitate the routine embryonic developmental process of the liver within a few days of *in vitro* culture. Most of the induction processes are ineffective, complicated, time-consuming, expensive, and limited by the difficulties involved in scaling up the procedures. Cytochrome P (CYP) activity in human ESC-Heps was much lower than in human primary hepatocytes cultured for 4 h and was stable or increased for at least one week in culture, which contrasts the observation of rapid loss of CYP activity in cultured human primary hepatocytes (Ulvestad et al., 2013). However, ESC-Heps are unable to function as efficiently as hepatoblasts or primary hepatocytes upon transplantation in liver repopulation models. Clearly, improvement of *in vitro* hepatic differentiation protocols and a better understanding of the molecular mechanisms underlying liver development are needed. Therefore, it can be speculated that there are certain specific TFs, or a combination of TFs, that can facilitate the differentiation of ESCs. Exposure of adherent human ESCs in culture to activin A treatment followed by various GFs, including dexamethasone, oncostatin M, and HGF, results in the production of ESC-Heps that possess hepatocyte-specific functions (Moore and Moghe, 2009). The signaling molecules bone morphogenic protein (BMP) and FGF that have been implicated in hepatic differentiation during normal embryonic development and have been shown to play pivotal roles in generating hepatic cells from DE cells derived from ESCs. Using a set of human adult markers, including CAAT/enhancer binding protein (C/EBPalpha), hepatocyte nuclear factor 4/7 ratio (HNF4alpha1/HNF4alpha7), CYP7A1, CYP3A4 and constitutive androstane receptor, and fetal markers, including AFP, CYP3A7, and glutathione S-transferase P1, by 21 days of differentiation, ESC-Heps have the characteristics of fetal hepatocytes at less than 20 weeks of gestation, but extending the differentiation to 4 weeks does not improve cell maturation (Funakoshi et al., 2011). Li et al. (Li et al., 2011a) established an efficient method for the induction of mouse ESC-derived DE cells in suspension embryonic body culture. The chemical activation of the canonical Wnt signaling pathway synergized with the activin A-mediated nodal signaling pathway to promote the induction of DE cells, and inhibition of BMP4 signaling by Noggin and activin A further improved the efficiency of DE cell differentiation. A combined treatment with Wnt3a and BMP4 efficiently differentiated human ESCs (Kim et al., 2013); after co-culture with STO feeder cells, human ESCs were able to differentiate into HLCs and cholangiocyte-like cells (Zhao et al., 2009). Forkhead box A2 (Liu et al., 2013) and synthesized basement membrane components (Shiraki et al., 2011) significantly increased the hepatic differentiation of ESCs.

Most studies demonstrating hepatic differentiation from ESCs have been based on embryoid body (EB) formation, aggregated colony formation in static culture. It has been shown that dynamic three-dimensional perfusion culture is superior to other culture systems for inducing maturation of ESCs into fetal hepatocytes and prolonging the maintenance of the hepatic functions of those cells. Ten pathways that were significantly upregulated in cells differentiated in a bioreactor compared to cells grown in static culture were shown to be highly related to liver functions (Sivertsson et al., 2013). The differentiated phenotype was sustained for more than 2 weeks in the three-dimensional spheroid culture system, which is significantly longer than in monolayer culture (Subramanian et al., 2014). EB-derived cells grown in a rotating bioreactor exhibited higher levels of liver-specific functions than those in static culture (Zhang et al., 2013a). The hollow fiber/organoid culture method allows for cultured ESCs to form an organoid, and the differentiating ESCs reach a level of functionality comparable to or better than that of primary mouse hepatocytes (Amimoto et al., 2011). Differentiated cells grown on a biodegradable polymer scaffold and a rotating bioreactor also exhibit morphologic traits and biomarkers characteristic of liver cells (Wang et al., 2012). Significantly upregulated hepatic gene expression was observed in the hepatic differentiation hollow fiber-based three-dimensional perfusion bioreactors with integral oxygenation culture group (Miki et al., 2011). However, most of the current three-dimensional differentiation configurations involve interruptive operations during the multistaged differentiation process, which might impose unwanted influence on cellular differentiation. Off-the-shelf micro-stencil arrays were developed to generate adherent multilayered colonies composed of human ESC-derived cells; the microscaled multilayered colonies with uniform and defined sizes constrained within the microwells are composed of more homogenous and mature HLCs with significantly lowered AFP expression and elevated hepatic functions (Yao et al., 2014). A combination of co-culture with non-parenchymal liver cells and optimal GF stimulation was found to induce endoderm and hepatic phenotypes earlier and to a much greater extent than GF arrays or micropatterned co-cultures used individually (Tuleuova et al., 2010). When coculturing ESCs with MLSgt20 cells, which are derived from mesenchymal cells residing in murine fetal livers, human ESC-derived AFP-producing cells displayed higher hepatocyte functions (Ishii et al., 2010). A similar effect was observed in mouse ESCs co-cultured with mouse HSCs as feeder cells in basal medium without additional hepatocyte growth factors (Chan et al., 2013).

#### *Induced pluripotent stem cell-derived hepatocytes* (*iPSC-Heps*)

iPSCs were initially generated from terminally differentiated adult cells using viral vectors for specific TFs, and later using the non-integrating methods of treating cells with small molecules that affect methylation or acetylation, mimic the Wnt-signaling pathway, or modulate the TGF-b pathway (Li et al., 2009). These methods raised few ethical concerns because of their derivation from somatic cells and, thus, are powerful tools for studying basic developmental biology. iPSCs are now considered to have the same level of pluripotency as ESCs (Jia et al., 2010), and the mitochondria of iPSCs were shown to act like those of ESCs by measuring oxygen concentration and pH in hepatic induction medium, which indicate the oxygen consumption rate and extracellular acidification rate, respectively (Tamai et al., 2011). iPSCs can be differentiated into neural, osteogenic, cardiac, adipogenic, pancreatic, vascular, hematopoietic, and endothelial cells. In addition, they are potential sources of hepatocytes for applications in regenerative medicine and drug development (Noto et al., 2014). iPSCs from mouse embryonic fibroblasts and human fibroblasts can be differentiated into hepatic lineages with four reprogramming factors (Oct-4/Sox2/Klf-4/c-Myc) (Yamanaka and Blau, 2010). Li et al. (Li et al., 2011b) demonstrated that iPSCs could be differentiated into iPSC-Heps with biological functions without being treated with c-Myc, and those iPSC-Heps reduced the hepatic necrotic area and improved liver function. However, iPSCs *in vivo* are able to create chimeras by germ line transmission and tetraploid complementation and form teratomas containing various cell types from three embryonic germ layers.

Liu et al. (Liu et al., 2010a) were the first to reprogram primary hepatocytes to pluripotency, and these hepatocyte-derived iPSCs were able to directly differentiate into DE, HPCs, and mature hepatocytes. The derivation of iPSCs from somatic cells of patients with liver diseases, including tyrosinemia, glycogen storage disease, progressive familial hereditary cholestasis, and Crigler-Najjar syndrome (two siblings), through retroviral transduction of Yamanaka’s factors in serum and feeder-free culture conditions has been shown (Ghodsizadeh et al., 2010). Furthermore, those iPSCs were efficiently differentiated into functional HLCs (Ghodsizadeh et al., 2010). iPSCs are specified to primitive streak/mesendoderm/definitive endoderm by sequential stimulation with liver development-related cytokines, resulting in differentiated cells with characteristics of HLCs. CYP activities in iPSC-Heps were stable or increased for at least one week in culture, which contrasts with the rapid loss of CYP activities in cultured human primary hepatocytes between 4 h and 48 h after plating (Ulvestad et al., 2013). iPSC-Heps can be directed to differentiate into HLCs by mimicking embryonic and fetal liver development (Hannan et al., 2013; Kajiwara et al., 2012; Takebe et al., 2013), but most differentiated cells co-express AFP and ALB, suggesting incomplete cell maturation. Although progress in developing differentiation procedures has been made, it remains challenging to generate iPSC-derived mature hepatocytes. Umeda et al. (Umeda et al., 2013) performed knock-in of a monomeric Kusabira orange (mKO1) cassette into the ALB gene in iPSCs with the use of a helper-dependent adenovirus vector, and the ALB/mKo1 knock-in iPSCs are valuable resources, as they show enhanced *in vitro* hepatic differentiation function.

Compared with the *in vivo* environment of the liver, culture conditions are relatively artificial, and this is likely to impact the function of iPSC-Heps (Si-Tayeb et al., 2010). Activin has the opposing effects of promoting differentiation into endoderm and maintaining pluripotency by regulating the expression of Nanog (Shin et al., 2011). Dexamethasone and insulin-transferrin-selenium are used to maintain the *in vitro* functions of hepatocytes. In consideration of the tedious differentiation work and complex combination of cytokines, many investigators have focused on developing concise and rapid methods for differentiating iPSCs into HLCs. Chen et al. (Chen et al., 2012) established a rapid and efficient three-step differentiation protocol with HGF, activin A, and Wnt3a that is able to generate functional HLCs from human iPSCs. Then, Takata et al. (Takata et al., 2011) described a two-step protocol for directing human iPSCs to differentiate into hepatic cells using only two cytokines and a short incubation time; furthermore, the differentiation efficiency of the two-step protocol was comparable to that of the three-step protocol, and the induced hepatic cells were functional. In the latest study by Tomizawa et al. (Tomizawa et al., 2013), a single-step protocol for the differentiation of iPSCs into hepatocytes was designed and involved exposure to FoxA2, GATA4, HEX, and C/EBPα and culturing with OERDITS supplementation. This protocol has the potential to induce the differentiation of iPSCs into HLCs within 8 days. Kondo et al. (Kondo et al., 2014) developed a simple method of differentiation of human iPSCs into functional HLCs with small-molecule compounds, which are convenient and inexpensive to obtain for large scale production and do not have the potential to be contaminated with exogenous viruses or cells. However, Zhang et al. (Zhang et al., 2014) showed that human iPSC-derived mature HLCs hardly ever proliferated *in vitro*, and in contrast, human iPSC-derived hepatic endoderm cells exhibited a marked proliferative capability.

All of the protocols discussed above are static culture protocols for iPSCs, and three-dimensional culturing protocols can significantly enhance the function of HLCs. Hollow fiber/organoid culture of mouse iPSCs to induce expression of liver-specific genes and functions allows for spontaneous differentiation with cell proliferation and self-organization, high cell density, and the induction of differentiation in a large number of cells (Amimoto et al., 2011). The micro-cavitary hydrogel (MCG) system enhances nutrient exchange, permits greater living space for the encapsulated pluripotent stem cells to rapidly grow into colonies and results in significantly greater production of endoderm markers, hepatic markers, urea, and ALB by iPSCs compared to the typical non-MCG system, or monolayer culture (Lau et al., 2013). Zhang et al. (Liu et al., 2013) reported three-dimensional clump culture collagen matrices compatible with high throughput screening resulted in significantly increased functional maturation of iPSC-Heps towards an adult phenotype when compared to conventional culture systems. Additionally, this approach spontaneously results in the presence of polarized structures necessary for drug metabolism and improves the functional longevity when culturing *in vitro* over 75 days. Chiang et al. (Chiang et al., 2015) reprogrammed human dental pulp-derived fibroblasts into iPSCs, developed an injectable carboxymethyl-hexanoyl chitosan hydrogel (CHC) with sustained HGF release and investigated the hepatoprotective activity of HGF-CHC-delivered iPSC-Heps *in vitro*. Compared with PBS-delivered iPSC-Heps, the HGF-CHC-delivered iPSC-Heps exhibited higher antioxidant and anti-apoptotic activities, which resulted in a reduction of the hepatic necrotic area. Chien et al. (Chien et al., 2015) demonstrated that embedment of miR122 complexed with a polyurethane-graft-short-branch polyethylenimine copolymer in nanostructured amphiphatic carboxymethyl-hexanoyl chitosan led to dramatically enhanced miR122 delivery into human dental pulp-derived iPSCs and facilitated these cells to differentiate into iPSC-Heps with mature hepatocyte functions.

#### *Hepatic progenitor cell-derived hepatocytes* (*HPC-Heps*)

The first HPCs were identified by Farber (1956) and were termed oval cells, a small bipotent cell type with a high nuclear-to-cytoplasmic ratio, but in human it was defined as HPCs. They have been shown to emerge in several human liver diseases, including primary biliary cirrhosis (Crosby et al., 1998), primary sclerosing cholangitis (Vessey and de la Hall, 2001), and hepatocellular adenoma (Libbrecht et al., 2001). PH-activated progenitor cells are hepatic stem-like cells that can be cultured *in vitro* for more than 3 months, with the number of cells doubling 100 times over that period (He et al., 2004). Human liver-derived stem cells can be isolated and expanded from donated livers unsuitable for transplantation and present a comparable morphology to that of HSCs, which express a-smooth muscle actin, vimentin, fibronectin, CD73, and CD90 in accordance with their mesenchymal origin (Berardis et al., 2014). They can be induced to differentiate into cells with morphological, phenotypic, and functional characteristics of mature hepatocytes (He and Feng, 2011). Moreover, HPCs have a greater regenerative capacity than adult hepatocytes and participate in liver tissue repair and reconstruction following injury. It has been reported that HPCs transplantation can be used as a substitute for liver transplantation, and HPCs have a definite therapeutic effect on patients with end-stage liver diseases (Hughes et al., 2012). The effect of different PHs and the duration of collagenase perfusion on hepatic stem cell proliferation and differentiation varies; optimal differentiation of hepatic stem cells to CK-18 and AFP-positive cells was observed when stem cells isolated from 83.4% PH rats were perfused with IV collagenase for 20 min (Gong et al., 2013). Thy1 and CD44 oval and progenitor cells are able to differentiate into hepatocytes, but the degree of maturation of the induced hepatocytes may not be equal to that of healthy resident hepatocytes (Ichinohe et al., 2013). Messenger RNA expression levels of CYP1A2, CYP2B1/2, and CYP3A1 were higher in cells of young rats, and the proliferation and differentiation potential of oval cells decreased with age (Czekaj et al., 2010). Wang et al. (Wang et al., 2010) demonstrated slight acceleration of proliferation of hepatic oval cells after the 50th passage, but the cells remained diploid with features of chromosomal stability. Furthermore, they did not acquire anchorage-independent growth capacity and did not develop into tumors in immunodeficient mice, suggesting that hepatic oval cells do not undergo spontaneous malignant transformation.

Current protocols for the differentiation of oval cells make use of multiple treatments of soluble signals and/or matrix factors and typically result in partial differentiation of oval cells to mature cells with under- or over-expression of adult tissue-specific genes. The activation of AKT, p70s6k, and ERK1/2 induced by HGF in OC/CDE22 rat oval cells was abolished by pretreatment with a phosphoinositide 3-OH kinase inhibitor and a mitogen-activated protein kinase/ERK kinase inhibitor, respectively (Okano et al., 2003). This finding suggested that this signaling pathway is responsible for the biological effect of HGF. LE/2 and LE/6 oval cells are non-tumorigenic cells that were derived from the livers of adult rats fed a choline-deficient diet containing 0.1% ethionin. After 4 weeks in a three-dimensional collagen gel matrix and a fibroblast feeder layer culture system, these cells acquired typical hepatocytic morphology; however in the absence of a feeder layer and in the presence of HGF and/or keratinocyte growth factor, the precursor cells formed ductal structures, suggestive of differentiation along the bile duct lineage (Lazaro et al., 1998). The hepatic stem cell lines HY1, HY2, and HY3, which were derived from healthy livers of adult rats, showed an expression pattern similar to oval cells and efficiently induced hepatic differentiation following sequential treatment with type I collagen, TGF-b1, and HGF or oncostatin M (Hirata et al., 2009). Human hepatic stem cells seeded onto liver biomatrix scaffolds in a hormonally defined medium tailored for adult liver cells lost stem cell markers and differentiated into mature, functional parenchymal cells and remained viable with stable mature cell phenotypes for more than 8 weeks (Wang et al., 2011).

#### *Mesenchymal stem cell-derived hepatocytes* (*MSC-Heps*)

MSCs are highly proliferative, adherent mesenchymal cells with a unique cell surface molecule expression profile. The differentiation potential of adult stem cells has long been believed to be limited to the tissue or germ layer of their origin. In addition to long-term self-renewal capability, MSCs possess versatile differentiation potential ranging from mesenchyme-related multipotency to neuroectodermal and endodermal competency. Furthermore, they have the ability to differentiate into a number of organ-specific cell types, including hepatocytes (Christ and Dollinger, 2011). Adult stem cells can be derived from different tissues, such as bone marrow, adipose, placenta, umbilical cord blood, menstrual blood, and synovial tissues, etc.

When MSCs are treated with an external factor, their differentiation ability may be improved. Cytokines may play a more important role in the differentiation of MSCs into hepatocytes. When HGF, nicotinamide, or dexamethasone was added to MSCs individually, incomplete hepatocyte differentiation was achieved; the obtained cell populations contained progenitors that expressed both hepatic (ALB) and biliary (CK19) markers as well as AFP. When all factors were added to the culture medium, the cells exhibited features that closely resembled human adult hepatocytes (Chivu et al., 2009). A combination of insulin-like growth factor-I and liver-specific factors supported the potential development of MSCs into primary hepatocytes (Ayatollahi et al., 2011), and the addition of dimethylsulfoxide enhanced their differentiation into hepatocytes (Seo et al., 2005). Alpha-1 antitrypsin (AAT) deficiency is a hereditary disorder characterized by a severe decrease in AAT plasma level, which leads to progressive liver dysfunction; however, human MSC-Heps can be AAT genetically modified as a novel paradigm of coupling cell therapy for this disease (Ghaedi et al., 2010). Acute hepatic failure-derived bone marrow mesenchymal stem cells (BMMSCs) have a hepatic transcriptional profile, express hepatocyte-specific genes early during differentiation, and possess greater hepatogenic potency *in vitro* compared to cells isolated from control animals (Li et al., 2010a). Pretreatment of MSCs with injured liver tissue in an *in vitro* model resulted in high expression of albumin, CK8, CK18, transaminase, and HNF1a compared to untreated MSCs, indicating that this pretreatment augmented the homing and hepatic differentiation abilities of MSCs (Mohsin et al., 2011). Culturing in hepatocyte-conditioned medium without any cytokines can induce the differentiation of BMMSCs into HLCs (Chen et al., 2007). Quiescent HSCs or culture-activated HSCs do not have the ability to modulate the differentiation of MSCs. Moreover, Kupffer cell-activated HSCs expressed HGF mRNA, and culture-activated HSCs did not (Deng et al., 2008).

MSCs grown on nanofibers showed enhanced differentiation into HLCs and maintained their function in long-term culture; hepatocyte markers ALB and HNF4α were elevated in a time-dependent manner, and CYP450 enzymes were significantly increased in the HLCs differentiated *in vitro* from MSCs grown on nanofibers at day 36 (Piryaei et al., 2011). BMMSCs cultured in alginate scaffolds in the presence of specific GFs display several liver-specific markers and functions (Lin et al., 2010). A three-dimensional co-culture system of porcine hepatocytes and BMMSCs was established *in vitro*, and the best hepatic function levels were achieved on day 2 and moderately decreased in the following co-culture days (Gu et al., 2009). Culturing human adipose-derived mesenchymal stem cells (ADMSCs) on top of HGF/Col spots (HGF co-printed with collagen I to create arrays of protein spots on glass) for 2 weeks can differentiate them into HLCs (Ghaedi et al., 2011). Floating culture efficiently induced human ADMSCs into functional HLCs *in vitro* (Okura et al., 2010).

## FUTURE DIRECTIONS

The liver regeneration process has been evaluated in PH models. Although it has been studied extensively, many important fundamental mechanisms remain undefined, such as the mechanisms of cellular hypertrophy, cell division, nuclear division, ploidy changes, and organ size control. The current shortage of donor organs available for live transplantation and the severe morbidity and mortality associated with this procedure underscores the need for alternatives to liver transplantation. *In vivo* hepatocytes proliferate to repair injured liver tissue, but this process is too complex to mimic *in vitro* to obtain functional hepatocytes. Culture methods for the enhancement of isolated primary hepatocytes or HLCs *in vitro* include the addition of GFs, TFs, and cytokines, the activation of signaling pathways, the use of an optimized matrix, and co-culture with various nonparenchymal cells. All of these methods are able to improve the function of primary hepatocytes or HLCs *in vitro* to a certain extent, but unfortunately, the amount of available mature hepatocytes have been insufficient for clinical use in the past several decades. And the lack of standardization of protocols for isolating specific cell types and the use of a variety of injury/disease models have made the interpretation of these results rather difficult and have left unanswered questions regarding the mechanisms through which these cells generate their beneficial effects.

The observed effects of cell therapies for liver regeneration have certainly generated reserved optimism toward their value for clinical use once the methodologies have become standardized and optimized. With clarification of the process of liver regeneration, hepatocytes or HLCs that can grow and proliferate persistently and functionally *in vitro* and *in vivo* will be able to be used to fulfill the needs of injured livers for regeneration, and cell transplantation and artificial liver support will completely replace liver transplantation.
